# Correlated Polymer‐Proton Hopping Enables Water‐Independent Fast Proton Transport in Poly(Ionic Liquid) Membranes

**DOI:** 10.1002/advs.77430

**Published:** 2026-08-29

**Authors:** Keenan Smith, Antonela Gallastegui, Zixuan Yu, Yuliana Pairetti, Andrew Seel, Jacques Ollivier, Victoria Garcia Sakai, Bob C. Schroeder, Maria Forsyth, Aurelie Gueguen, David Mecerreyes, Fabrizia Foglia

**Affiliations:** ^1^ Department of Chemistry University College London London UK; ^2^ POLYMAT University of the Basque Country UPV/EHU Donostia‐San Sebastian Spain; ^3^ ISIS Neutron and Muon Source Rutherford Appleton Laboratory Harwell Science and Innovation Campus Chilton UK; ^4^ Department of Physics Royal Holloway University of London, Egham Hill Egham UK; ^5^ Institut Laue Langevin Grenoble France; ^6^ IKERBASQUE Basque Foundation for Science Bilbao Spain; ^7^ Material Engineering Division Toyota Motor Europe NV/SA Zaventem Belgium

**Keywords:** fuel cell, ionic liquid, neutron reflectivity, PFG NMR, proton transport, QENS, SAXS

## Abstract

Polymer electrolyte membranes composed of ionic liquids (IL), capable of conducting protons efficiently at elevated temperatures, without external humidification, could transform fuel cell technology. However, the molecular origins of such proton transport remain poorly understood, especially in the polymerized state. Here, we directly visualize a hierarchy of coupled elementary proton motion steps spanning picosecond to nanosecond timescales in polyIL membranes using pulse field gradient (PFG) NMR and multi‐resolution quasi‐elastic neutron scattering (QENS). Polymer dynamics comprise three‐site jumps within methanesulfonate coordination shells, two‐site hops along hydrogen‐bond chains, and out‐of‐plane backbone flips which dynamically reconfigure the proton transfer pathway. The latter facilitates a correlated polymer‐proton hopping mechanism above 60°C enabling rapid nano‐ and microscale proton transport at operational temperatures. Even trace water plasticizes the polymer and remarkably lowers this proton hopping barrier by nearly half. A critical transition occurs near 245 K, where water forms a continuous hydrogen‐bonded network of acid‐base pairs, enabling sub‐10 ps Grotthuss proton hopping, approaching liquid water dynamics. This molecular‐level understanding provides fundamental insight into polyIL conduction mechanisms and the long‐standing question of how solid polymers achieve liquid‐like proton mobility, providing a roadmap for polyILs in next‐generation electrochemical energy technologies.

## Graphical abstract

A polymer chain in motion: backbone, ring, and counterion dynamics interlock across timescales to relay protons through poly(ionic liquid) membranes.

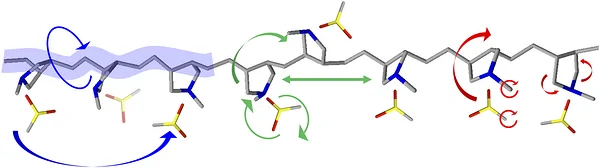

## 1 Introduction

Proton exchange membrane fuel cells (PEMFCs) provide a clean and efficient route for converting hydrogen into electricity and are central to decarbonizing transport and stationary power systems [1, 2, 3, 4]. Operating PEMFCs at 100–200°C would transform performance, accelerating both hydrogen oxidation and oxygen reduction kinetics, reducing reliance on platinum‐group catalysts, improving heat rejection, and increasing tolerance to CO and impure hydrogen feeds [5, 6]. Realizing these benefits, however, requires PEMs that retain high conductivity under low‐humidity or anhydrous conditions.

Perfluorosulfonic acid (PFSA) membranes such as Nafion conduct protons through water‐filled nanochannels via vehicular diffusion, in which protons move as hydronium ions (H_3_O^+^), or the Grotthuss mechanism, involving proton hopping through hydrogen‐bonded water chains [7, 8]. Although conductivities exceed 0.1 S cm^−1^ near 80°C [9], dehydration above 100°C collapses the hydrogen‐bond network, while the high cost, complex humidification requirements, and environmental persistence of fluorinated materials motivate alternatives [10]. Phosphoric acid‐doped polybenzimidazole membranes currently define the state of the art for high‐temperature PEMFCs [11]. Phosphoric acid serves as the mobile proton carrier, enabling conductivities of 50–100 mS cm^−1^ at 150–180°C [12]. However, high acid loadings weaken its structure and leaching can contaminate catalysts, motivating the search for intrinsically proton‐conductive membranes that do not rely on leachable dopants [13, 14, 15].

Ionic liquids (ILs) offer an attractive route owing to their negligible vapor pressure, high thermal stability, and intrinsic ionic conductivity [16, 17]. Protic ionic liquids (PILs) contain transferable protons that directly mediate charge transport [18, 19]. Their proton conductivity depends on the acid‐base strength difference: large values yield fully dissociated moieties, while smaller ones produce mixed ionic‐neutral systems where hydrogen‐bond networks enable hopping [20, 21, 22]. While PILs combine high conductivity and thermal resilience, their liquid nature hinders integration into membrane electrode assemblies (MEAs). Poly(ionic liquid)s (PolyILs) overcome this limitation by covalently tethering IL units into polymer backbones or side chains, combining the conductivity of ILs with the mechanical robustness of solid polymers [23]. Nanophase separation produces continuous ionic domains that function as solid‐state conduction channels, offering a promising path toward durable, high‐conductivity membranes for next‐generation high‐temperature PEMFCs [24].

Polymerization fundamentally alters ion transport in polyILs. Covalent tethering of ionic groups restricts long‐range translational motion, making conductivity dependent on the coupling between polymer segmental dynamics and short‐range ion hopping [25, 26]. The glass transition temperature (*T_g_*) is therefore critical; above *T_g_*, segmental motion facilitates ion mobility, whereas below *T_g_*, rigidity suppresses conductivity [27]. Polymer architecture, spacer flexibility, and counterion chemistry collectively govern *T_g_* and local ion dynamics in this fixed geometry [28]. Weakly coordinating fluorinated anions such as bis(trifluoromethane)sulfonimide (TFSI^−^) or triflate TfO^−^ promote vehicular transport and lower *T_g,_* while more basic sulfonates, e.g., methanesulfonate (MsO^−^), provide a more optimal pKa differential and thus enhance hydrogen bonding and favor Grotthuss‐type hopping [27, 29, 30].

Among protic polyILs, poly(diallylmethylammonium) (poly(DAMAH^+^)) has emerged as a particularly promising system [31]. DAMA forms flexible backbones densely populated with cationic sites, creating membranes that combine high ionic conductivity with excellent mechanical and thermal stability. In particular, poly(DAMAH^+^ MsO^−^) membranes are readily processable, maintain strong proton transport under both humidified and anhydrous conditions, and show outstanding chemical robustness, without acid leaching or degradation [31]. These physical properties, in addition to a completely fluorine‐free chemistry, position DAMA‐based polyILs as leading candidates for next‐generation proton‐conducting membranes in intermediate‐ and high‐temperature PEMFCs, as well as battery electrode binders and electrolytes [32].

Despite these advances, the molecular origins of proton transport in polyILs remain poorly understood. Proton conduction arises from multiple coupled motions spanning wide timescales, and understanding how these motions cooperate is key to designing membranes that balance conductivity, mechanical strength, and durability. Conventional techniques such as electrochemical impedance spectroscopy and dielectric relaxation spectroscopy provide valuable macroscopic or long‐timescale information but lack the temporal and spatial resolution to capture elementary proton‐transfer events that govern conductivity. Quasi‐elastic neutron scattering (QENS) uniquely probes atomic‐scale motions on picosecond to nanosecond timescales, directly accessing the length‐ and time‐scales relevant to proton exchange, local hopping, and polymer relaxations [33, 34].

Previous QENS studies on PILs have revealed ultrafast proton dynamics even in the solid state [35], corresponding to a localized sub‐picosecond proton transfer step and a 20 ps rate‐limiting reorientation step in addition to long‐range diffusional processes [36]. However, systematic investigations of polymerized analogues, where ionic motion couples to polymer relaxations, have remained scarce, leaving a fundamental gap in understanding how solid polyILs achieve liquid‐like proton mobility.

**FIGURE 1:**
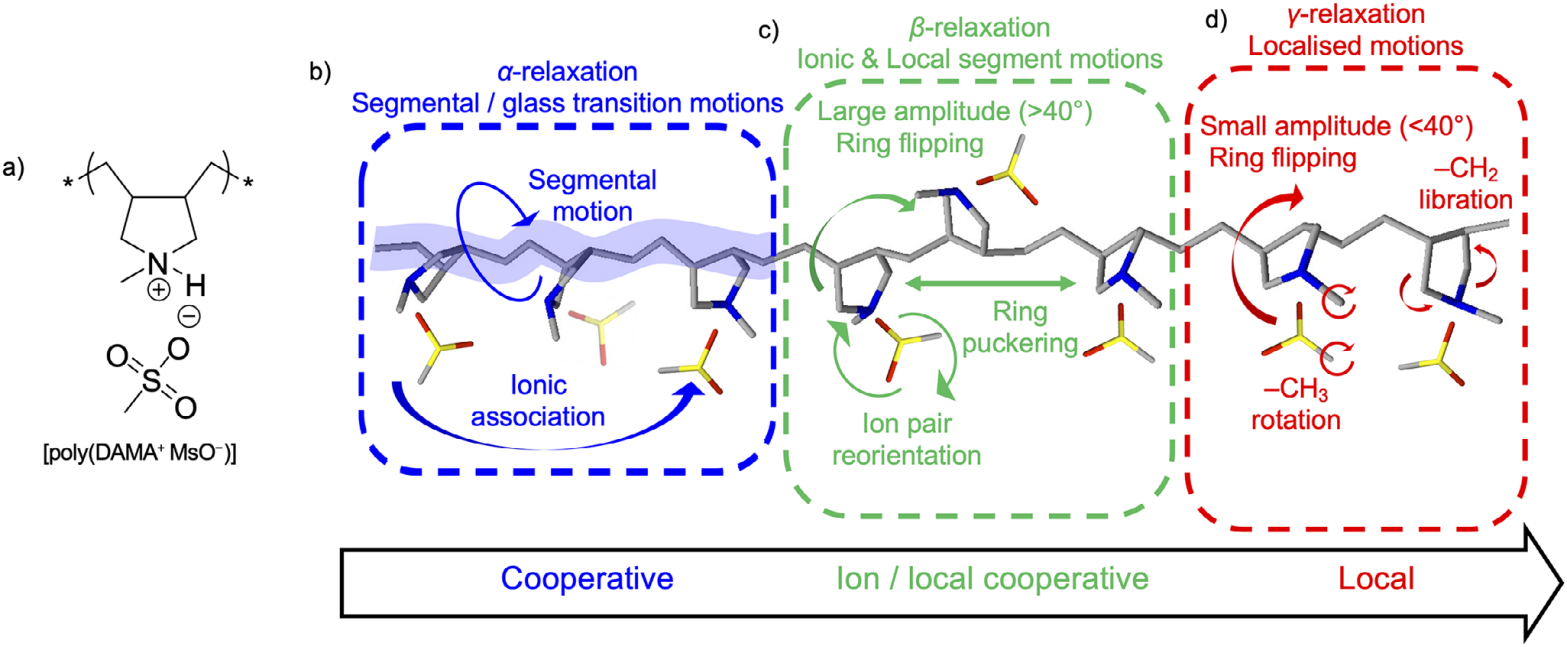
a) Molecular structure of poly(DAMAH^+^ MsO^−^). Hierarchy of relaxation dynamics in ionomer membranes across cooperative‐to‐local length scales. b) The *α*‐relaxation encompasses large‐scale segmental motion and ionic association, characteristic of segmental/glass transition dynamics. b) The *β*‐relaxation involves ionic and local segment motions, including large‐amplitude (>40°) ring flipping, ring puckering, and ion pair reorientation. c) The *γ*‐relaxation captures localized motions, including small‐amplitude (<40°) ring flipping, –CH_2_ libration, and –CH_3_ rotation. The progression from left to right reflects the transition from cooperative to purely local dynamic processes.

In this work, poly(DAMAH^+^ MsO^−^) (Figure 1a) was synthesized and characterized to elucidate the molecular origins and mechanistic pathways of solid‐state proton transport under systematic variation of temperature and hydration. Interplay of polymer segmental relaxation (i.e., *α*‐relaxation; Figure 1b) associated with backbone mobility and side‐chain mobility (i.e., *β*‐ and *γ*‐relaxation; Figure 1c,d, respectively), water molecular diffusion and localized proton hopping mechanisms were resolved by combining multi‐resolution QENS and ^1^H PFG NMR over picosecond to microsecond timescales. Complementary small and wide‐angle X‐ray scattering and neutron reflectivity were employed to probe nanoscale organization, water uptake, and swelling behavior. Together, these techniques enabled direct correlation between polymer dynamics, hydration, and proton transport in protic polyIL membranes.

## 2 Results and Discussion

**FIGURE 2:**
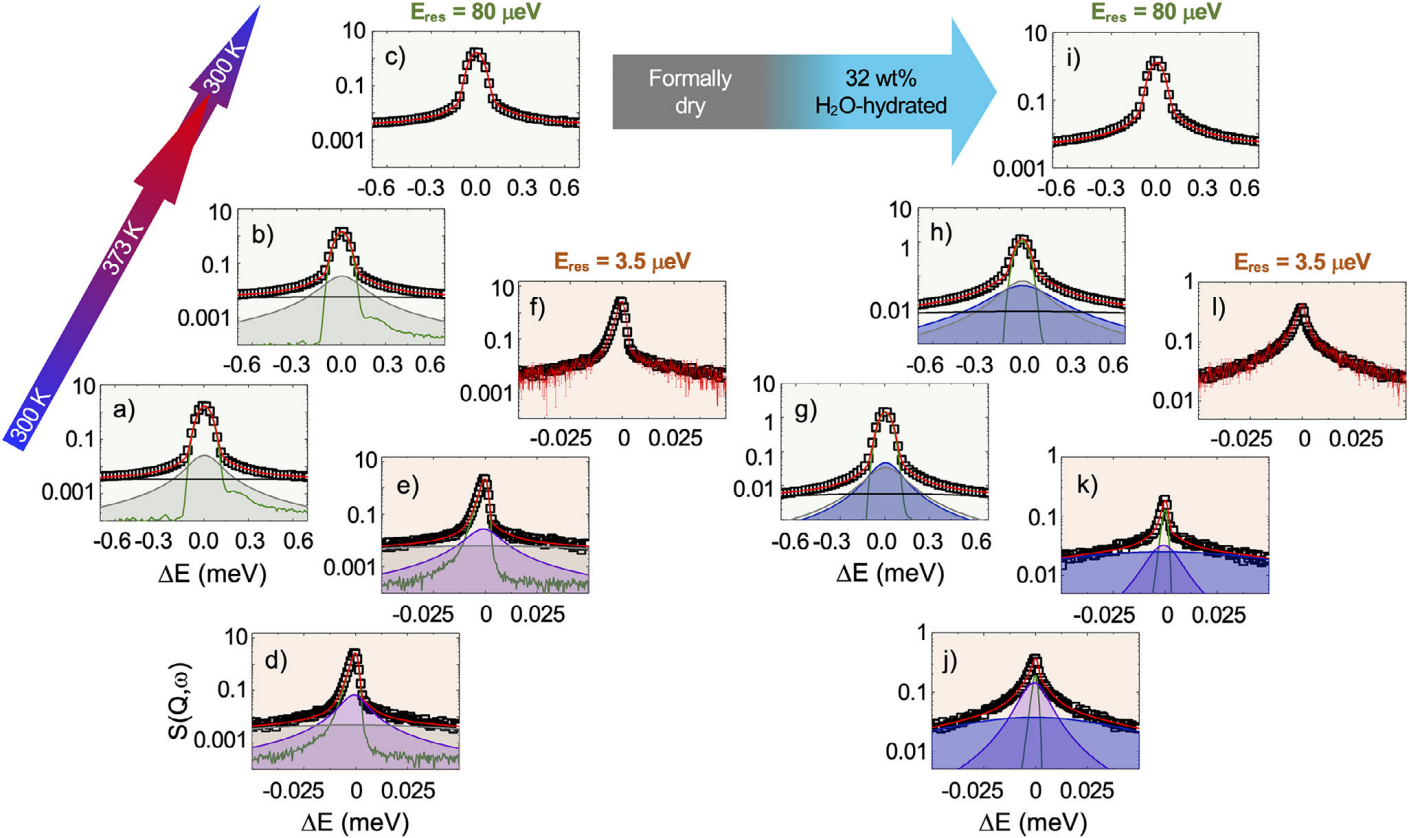
Multi‐resolution QENS on increasing temperature from 300 to 373 K, with *S(Q, ω)* experimental data in black squares, global fit in red, instrument resolution in green and Lorentzian fits of polymer dynamics in grey, hopping in purple and water in blue. IN5 (*E_re_*_s_ = 80 µeV; panels a–f) and BASIS (*E_res_* = 3.5 µeV; panels g‐l) fits for the dry (panels a‐c and g‐i, respectively) and the H_2_O‐hydrated state (32 wt.%; panels d‐f and j‐l; respectively). Material reversibility highlighted by overlaying 300 K *S(Q, ω)* data before and after 373 K heating are reported in panels c and i and f and l for formally dry and hydrated samples, respectively.

We employed multi‐resolution QENS (Figure 2 and Figure S1) by utilizing two complementary instruments, each providing unique insights into different aspects of the complex proton transport mechanism. The IN5 spectrometer (time‐of‐flight; ILL, France, Figure 2a–c,g–i) offers substantially broader energy coverage (∼0.5 ≤ *τ* ≤ 20 ps; *E_res_* = 80 µeV), with enhanced sensitivity to fast, localized dynamical processes including molecular vibrations, librational motions, and short‐range hopping events. Complementarily, the BASIS spectrometer (backscattering; SNS, USA, Figure 2d–f,j–l) provides ultrahigh energy resolution (∼100 ps ≤ *τ* ≤ 3 ns; *E_res_* = 3.5 µeV), which is specifically optimized for resolving slow molecular motions occurring in the nanosecond regime. This exceptional resolution enables precise quantification of long‐range diffusive processes and collective polymer relaxation dynamics that dominate at longer timescales.

QENS measurements were conducted over a broad temperature range (5–373 K), capturing multiple dynamical transitions: the frozen glassy state at cryogenic temperatures where only quantum tunnelling and restricted librations occur; the polymer glass transition (∼340 K) where cooperative segmental relaxation becomes activated; the ambient regime relevant to conventional fuel cell operation; and the elevated temperatures characteristic of high‐temperature PEMFCs. Complementary parallel studies were performed on D_2_O‐hydrated membranes at equivalent water content to selectively isolate and emphasize scattering signals originating from polymer backbone and MsO^−^ counterion dynamics.

**FIGURE 3:**
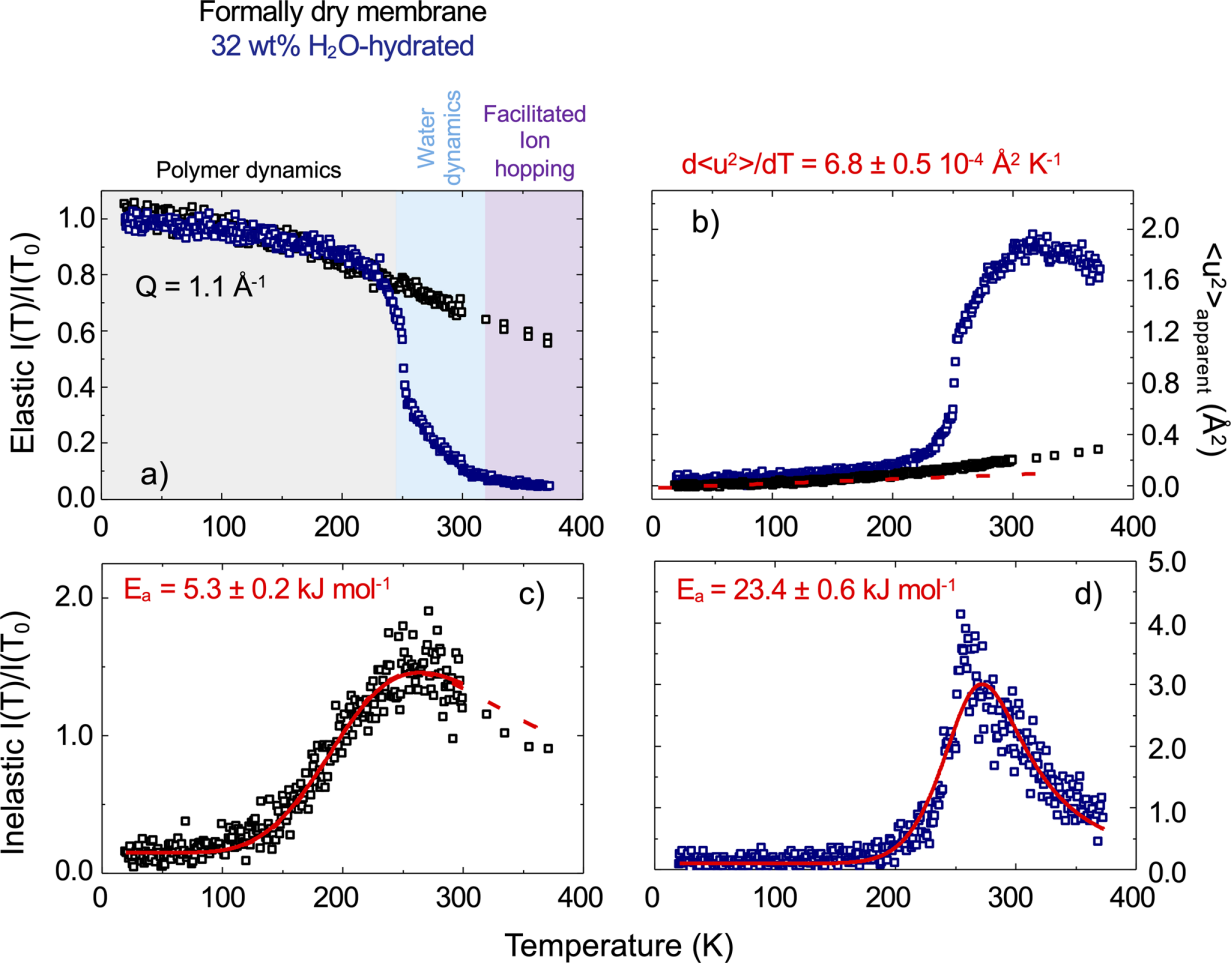
a,b) EFWS (Elastic Fixed Window Scan; panel a [31]) and apparent mean square displacement (m.s.d., *<u^2^>_apparent_*; panel b) as a function of temperature for formally dry (black squares) and hydrated (blue squares) poly(DAMAH^+^ MsO^−^) membranes collected on BASIS. The m.s.d. slope extrapolated from the data at low‐*T* is shown as a red dashed line. c,d) Corresponding IFWS (Inelastic Fixed Window Scan) of summed *Q*‐ranges of dry (panel c) and 32 wt.% hydrated polymer (panel d). Peak fits in red provide *E_a_* of the dynamic process [37]. The colored regions highlight three dynamic ranges, from left to right: 1) polymer dynamics, 2) water dynamics, and 3) facilitated ion hopping.

To systematically characterize the temperature‐dependent evolution of molecular dynamics and identify critical transition temperatures associated with the onset of different motional processes, we initially performed Elastic Fixed Window Scan (EFWS) measurements (Figure 3a and Figure S2). This technique monitors the integrated elastic scattering intensity as a function of temperature while maintaining constant energy resolution, thereby providing quantitative information about the population fraction of atoms or molecular segments that remain effectively immobile on the experimental observation timescale defined by the instrumental resolution. The complementary evolution of quasi‐elastic scattering intensity was simultaneously characterized through Inelastic Fixed Window Scan (IFWS) analysis (Figure 3c,d). The IFWS directly tracks how molecular dynamics become progressively activated across different temperature regimes, revealing both the onset temperatures for specific motional processes and their characteristic activation energies [37].

For nominally dry polymer samples, the elastic intensity exhibits a gradual, monotonic decrease with increasing temperature, reflecting the progressive thermal activation of polymer relaxation dynamics. This behavior is characteristic of glass‐forming polymeric materials and follows approximately Arrhenius‐type temperature dependence at moderate temperatures (e.g., for *T < T_g_* [38]). Above *T_g_*, however, the data must be described by the Vogel‐Fulcher‐Tammann (VFT) model, which accounts for the cooperative nature of segmental dynamics and the associated free volume expansion that facilitates ion transport [39].

However, a particularly pronounced and distinct inflection point in the hydrated membrane EFWS is clearly observed in the intermediate temperature, centered around 270 K. This characteristic inflection, which manifests as an abrupt change in the temperature derivative of the elastic intensity, corresponds precisely to the onset temperature window where both water molecular dynamics and polymer backbone cooperative motions become simultaneously activated and contribute measurably to the overall quasi‐elastic scattering signal.

Quantitative analysis of the temperature‐dependent elastic intensity in this transition region yields a relatively modest activation energy of *E_a_dry‐segment_* = 5.3 ± 0.2 kJ mol^−1^. This activation energy is significantly lower than typical covalent bond rotation barriers (∼12 kJ mol^−1^ [40, 41] but comparable to hydrogen bond reorganization energies.

This value lies well below those typically reported for *γ*‐relaxations in polymer systems, which are generally in the range of 20–40 kJ mol^−1^ [42, 43] and attributed to conformational changes of pendant side groups (Figure 1d). This unusually low activation energy is, however, entirely consistent with highly localized methyl group rotation, a motion well characterized by QENS in analogous polymer systems (∼5 kJ mol^−1^ [44, 45]). In poly(DAMAH^+^ MsO^−^), two chemically distinct methyl groups are present as candidate sites for this motion: the N‐methyl group of the pyrrolidinium‐like ring, and the methyl group of the mesylate counterion. Both groups are small, spatially restricted, and subject to minimal steric hindrance, conditions for which such an activation energy is physically reasonable. It is also worth noting that methyl group rotation, by virtue of the large number of hydrogen atoms involved in this motion, dominates the incoherent neutron scattering signal. Any contribution from slower ring dynamics, such as pyrrolidinium ring flapping, which would be expected to carry a higher activation energy, may be masked by the intense methyl signal. We therefore assign this process to fast, localized alkyl group reorientation; more precisely, methyl group rotation or analogous fast local reorientation dynamics of small pendant groups, rather than to a classical *γ*‐relaxation involving conformational rearrangement of a longer pendant chain. This interpretation is consistent with the gradual onset of quasielastic broadening beginning near 100K (Figure 3c), as commonly observed for thermally activated methyl‐group rotation [44, 45].

Importantly, this QENS‐derived activation energy of ∼5 kJ mol^−1^ must be interpreted with care in the context of ionic conductivity. When considering the activation energy calculated from conductivity measurements (Figure S3 and Table S1), analysing the entire T‐range yields a value of 63.1 kJ mol^−1^, which represents an artefact.

Separating the glassy and rubbery regimes yields, below *T_g_*, a corrected Arrhenius activation energy of 81.1 kJ mol^−1,^ which is consistent with anion hopping in rigid‐backbone polyILs. Above *T_g_*, where the VFT description holds, the apparent activation energy decreases strongly with temperature as cooperative segmental motion becomes available.

The QENS value is an order of magnitude smaller than this corrected activation energy, immediately excluding any mechanistic link between the fast local dynamics and conductivity, as they operate on entirely different time and length scales. Instead, the measured conductivity likely reflects the combined contribution of proton transfer, localized ionic rearrangements, and anion mobility within the polymer matrix.

Hydration profoundly modifies this response. Even at 5 wt.%, the elastic intensity exhibits a sharper decline and shifts to lower temperature (∼200–245 K) induced by the presence of nanoconfined water that becomes mobile and plasticizes the polymer [5, 46]. In highly hydrated conditions (i.e., >20 wt.%), a pronounced drop in intensity occurs below 250 K, signalling the activation of bulk‐water‐mediated dynamics. Above this temperature, water molecules transition from localized librational motions to translational diffusion via extended hydrogen‐bonded networks that facilitate rapid proton transfer. The systematically lower elastic intensity of hydrated samples across all temperatures confirms enhanced molecular mobility relative to the dry polymer.

The elastic incoherent structure factor (EISF) represents a powerful analytical tool that provides direct quantitative information about the spatial geometry and characteristic length scales of localized molecular motions. The EISF is the ratio of elastic scattering intensity to total scattering intensity and exhibits characteristic *Q*‐dependence that directly reflects the underlying geometry of molecular motion. Different motional models produce distinctive EISF signatures that enable identification of the dominant dynamical processes.

**FIGURE 4:**
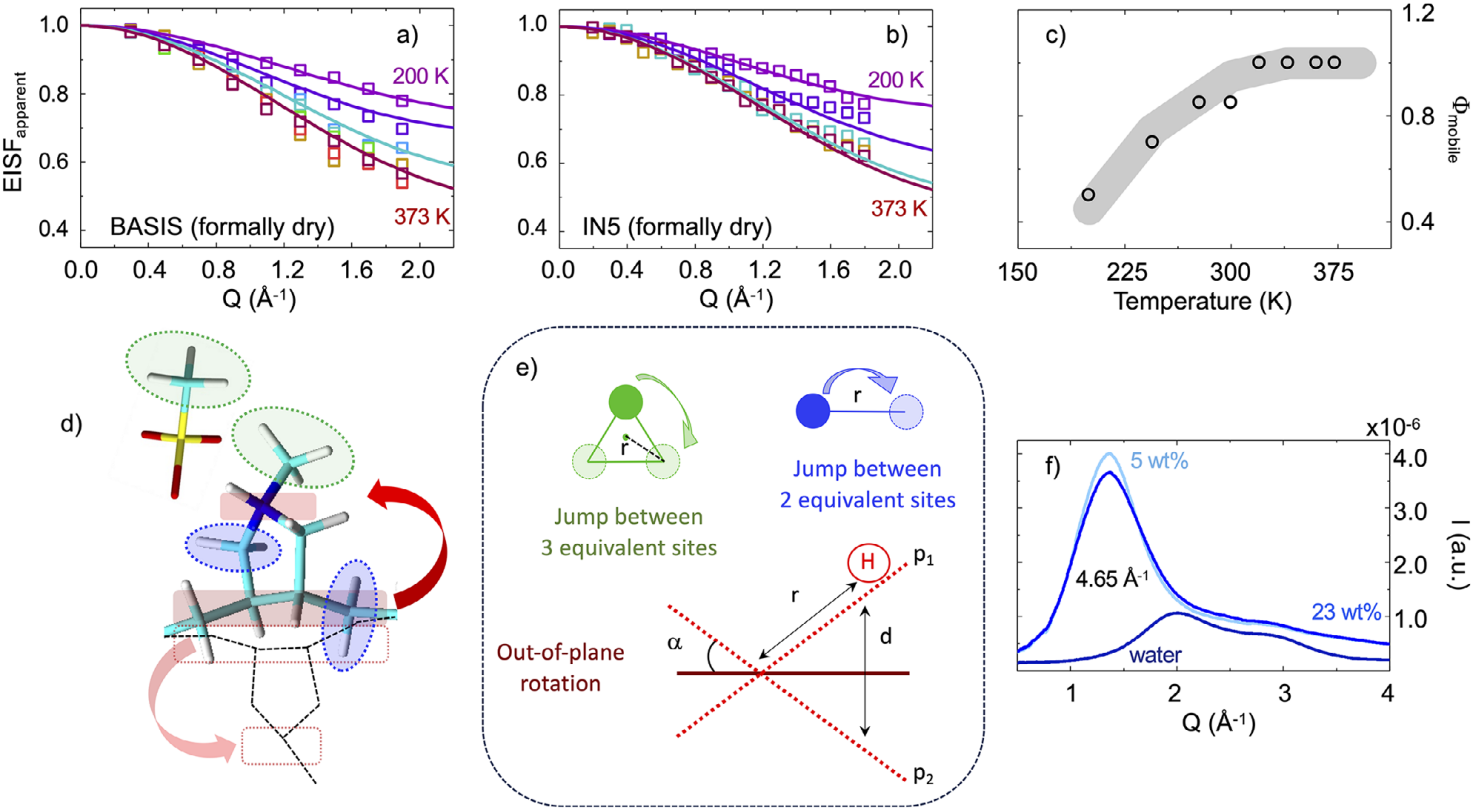
Geometry and populations of localized proton motions in poly(DAMAH^+^ MsO^−^) derived from QENS analysis. a,b) EISF as a function of *Q*, and fit accounting for the fraction of mobile protons across increasing temperature obtained from BASIS and IN5 data (panel a and b, respectively). c) Mobile proton fraction undergoing the three modelled dynamic modes in H_2_O (circles) and D_2_O (grey line) hydrated polymer. d) Schematic of the chemical structure and three proposed distinct motions; e) three‐site jumps within MsO^−^ and ammonium methyl, two‐site hopping of main chain –CH_2_ groups, and out‐of‐plane rotation (r = 2.8 Å). f) Wide‐angle X‐ray scattering (WAXS) of hydrated polymer and pure water reference. Data are reported for water (dotted line); polymer hydrated at 5 (light blue line) and 23 wt.% (dark blue line).

To ensure robust validation of the extracted motional parameters and exclude any possibility of spurious multimodal dynamics arising from instrumental artifacts or systematic errors, the EISF analysis was performed independently on data acquired from both neutron spectrometers (Figure 4a,b, respectively), leveraging their complementary *Q*‐ranges and energy resolutions. The remarkable consistency of EISF curves obtained from both instruments provides strong validation of the analytical methodology and confirms the intrinsic nature of the observed dynamical processes. EISF studies performed from 200 to 373K exhibit an equivalent, increasing fraction of mobile protons with temperature, at both resolutions (Figure 4c). Theoretical models representing different possible motional geometries for protons to participate with different fractional weighting were fit to the experimental EISF data. Three distinct and physically meaningful dynamical processes were definitively identified and quantitatively characterized.

- Three‐site jump process associated with the ‐CH_3_ rotation (highlighted in green in Figure 4d,e [47]).
- Two‐site jump process associated with the ‐CH_2_ (highlighted in blue in Figure 4d,e [47]).
- Out‐of‐plane rotational motion (Figure 4d,e [48, 49]) between two positions (p_1_ and p_2_) of equal proton populations with a characteristic radius, r = 2.8 Å and spatial displacement, d = 3.0 ± 0.3 Å.

The latter motional mode, which exhibits EISF oscillations consistent with rotational diffusion about a fixed axis, is attributed to librational motion in the form of a DAMAH^+^ unit flip around the main chain axis. This dynamic between energetically equivalent sites provides a symmetric population distribution. WAXS (Figure 4f) reveals the presence of a single amorphous halo peak due to the monomeric DAMA repeat units with an average nearest neighbor spacing of 4.6 Å in the amorphous matrix [27]. A lack of aromaticity and π stacking provides high polymer freedom in the disordered phase, and a ∼3 Å length scale corresponds well to the cation size, providing strong structural support for this assignment.

**FIGURE 5:**
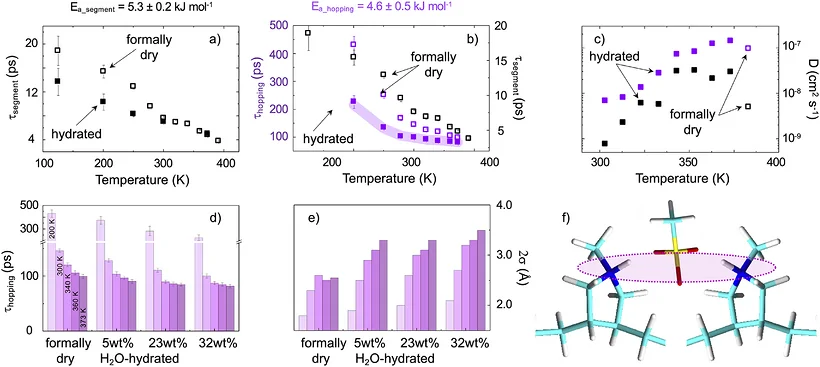
a) Polymer relaxation times (*τ_segment_*) for the polymer dynamics extracted from the QENS line‐widths for dry and 32 wt.% hydrated samples between *T*  =  120 and 393 K. b) Proton hopping timescale (*τ_hopping_*, purple) and *τ_segment_* (black) extracted from QENS line‐widths as a function of temperature between *T*  =  170 K and 373 K; D_2_O data are represented as shaded line. c) Diffusion coefficients of labile proton (purple) and polymer (black) extracted from ^1^H PFG NMR obtained at 5 wt.% hydration (filled square; *D_hydrated‐segment_*) and dry (open square; *D_dry‐segment_*) from 303 to 383 K. d‐e) *τ*_hopping_ (panel d) and hopping distance (*2σ*; panel e) *vs* temperature and H_2_O water uptake. f) Schematic of polymer with highlighted region where proton hopping occurs between anion and cation units.

By fitting to appropriate dynamical models, the temperature‐dependent quasi‐elastic broadening provides characteristic relaxation times for polymer dynamics (Figure 5). The characteristic relaxation time (*τ_segment_*; Figure 5a,b) is associated with local segmental motions spanning approximately from ∼20 to ∼5 ps (observed on IN5 between 125 and 373 K, respectively). The temperature‐dependent acceleration at ∼300 K is consistent with the inflection in the elastic intensity curves and fraction of mobile protons (Figures 3 and 4c), indicating a γ‐relaxation of the DAMAH^+^ unit which permits cation flipping within the polymer (Figure 3c).

A particularly noteworthy observation emerges from comparison of dynamics in dry versus water‐swollen membranes (Figure 4a); membrane swelling induced by water uptake substantially accelerates polymer relaxation dynamics. This plasticization exhibits pronounced temperature dependence with maximum impact at lower temperatures, consistent with the increasingly decoupled transport behavior commonly observed as ionic systems approach the glass transition regime [25]. Specifically, at 125 K water incorporation accelerates polymer relaxation by ∼30% relative to the dry state (*τ*_segment___dry_ = 19 ps *vs. τ*_segment___hydrated_ = 14 ps). At higher temperatures, polymer chains possess adequate intrinsic mobility, even in the absence of water, such that water‐induced free volume enhancement produces only marginal further acceleration. This temperature‐dependent behavior finds broader parallel in ionic liquid systems, where mobility‐enhancing effects are similarly most pronounced near the glass transition temperature. In pure imidazolium ionic liquids, an anomalous viscosity decrease upon quenching to near‐*T_g_* temperatures has been documented, with the effect being most pronounced closest to *T_g_* [50]. Similarly, polymerized ionic liquids exhibit approximately two orders of magnitude higher ion mobility than pure IL counterparts at the same *T/T_g_*, with this enhancement growing as temperature approaches *T_g_* [51]. These observations reinforce the principle that mobility‐enhancing effects are inherently most impactful where intrinsic molecular mobility is most severely constrained.

Superimposed upon, and intrinsically coupled to this *γ*‐relaxation, a distinct and slower dynamical process associated with proton hopping along the ionic network is activated. The relaxation time (*τ_hopping_*; Figure 5d) decreases from 430 to ∼100 ps (at 125 and 373 K in the dry membrane, respectively), with hopping proceeding via direct DAMAH^+^‐MsO^−^ ion‐pair proton transfer (Figure 5f): the single proton on nitrogen of DAMAH^+^ and three methyl protons on MsO^−^ constitute the four slow‐proton population identified in EISF analysis. These *τ_hopping_* values are systematically longer than *τ_segment_*, indicating that proton hopping constitutes the rate‐limiting step for overall charge transport rather than polymer reorganization. Further evidence emerges from the analysis of the hopping distance (*2σ* ∼3 Å, Figure 5e), which precisely matches the optimal X···H···O separation for strong, linear hydrogen bonds that enable barrierless or near‐barrierless proton relay.

This process exhibits an activation energy of *E_a_hopping_* = 4.6 ± 0.5 kJ mol^−1^, markedly lower than the typical proton‐transfer barriers observed in strongly hydrogen‐bonded systems (10–30 kJ mol^−1^ [52, 53]). Such an exceptionally low value aligns with those reported for packed‐acid and short‐range Grotthuss‐type conduction mechanisms (2–5 kJ mol^−1^ [54]), strongly indicating the presence of highly efficient, water‐independent proton conduction pathways. These ultralow barriers imply precisely organized hydrogen‐bond geometries that drastically reduce the energetic cost of proton transfer.

The activation energy associated with the hopping, calculated from the relaxation time, is 4.6 kJ mol^−1^, a value that bears a close correspondence to the activation energy of polymer relaxation (5.3 kJ mol^−1^). It is important to note, however, that the signal from which the hopping activation energy is extracted likely contains overlapping contributions from –CH_3_ group rotation and ion hopping, both low‐barrier processes whose superposition is consistent with the observed value, with ring flipping contributing only marginally to a minor extent. This spectral ambiguity notwithstanding, the correspondence between the two activation energies remains physically meaningful and uncovers a fundamental dynamic coupling between polymer relaxation and proton transport. Polymer segmental motions are not merely passive in this picture: DAMA unit ring flipping actively reshapes the local hydrogen‐bond topology [36], continuously regenerating optimal proton‐transfer routes and preventing kinetic trapping in suboptimal configurations. The overall result is a cooperative dynamic in which polymer flexibility and proton mobility are intrinsically coupled, reminiscent of self‐sustained proton transport in packed‐acid systems, even if the precise decomposition of the relaxation signal into its individual contributing motions requires further resolution.

Comparing QENS (ps‐ns), NMR (µs), and conductivity (ms) data provides a complete multiscale description of the conduction process. By fitting the ^1^H PFG NMR signal (Figure S4), using the Stejskal‐Tanner equation, two distinct diffusion coefficients were obtained, indicating contributions from different motional components in the system. These were associated with a labile H^+^ (i.e., the previously described proton which undergoes facilitated acid‐base hopping) and polymer backbone dynamics. Both dynamics exhibit similarly low diffusion coefficients and high activation energy below 340 K, above which a sharp increase occurs for labile H^+^ and a step‐like increase for the polymer backbone (Figure 5c). DSC measurements (Figure S5) demonstrate that hydration is a critical determinant of the thermomechanical behavior of poly(DAMAH^+^ MsO^−^). At fuel cell operating temperatures, the dramatic enhancement in segmental mobility unlocks the cooperative long‐range proton transport pathways essential for device performance [23]. The latter corroborates the decreasing *τ_segment_* and a plateau in hopping distance (*2σ*) above 340 K due to a thermally activated process operating over fixed geometry and tight coupling between segmental dynamics and proton mobility (Figure 5).

This spatial organization, combined with the low activation energy, provides compelling molecular‐level evidence that the polyIL architecture inherently promotes efficient, water‐free proton transport. Such behavior represents a critical advance for high‐temperature fuel cell applications, where eliminating water dependence mitigates one of the major limitations of conventional proton conductors.

**FIGURE 6:**
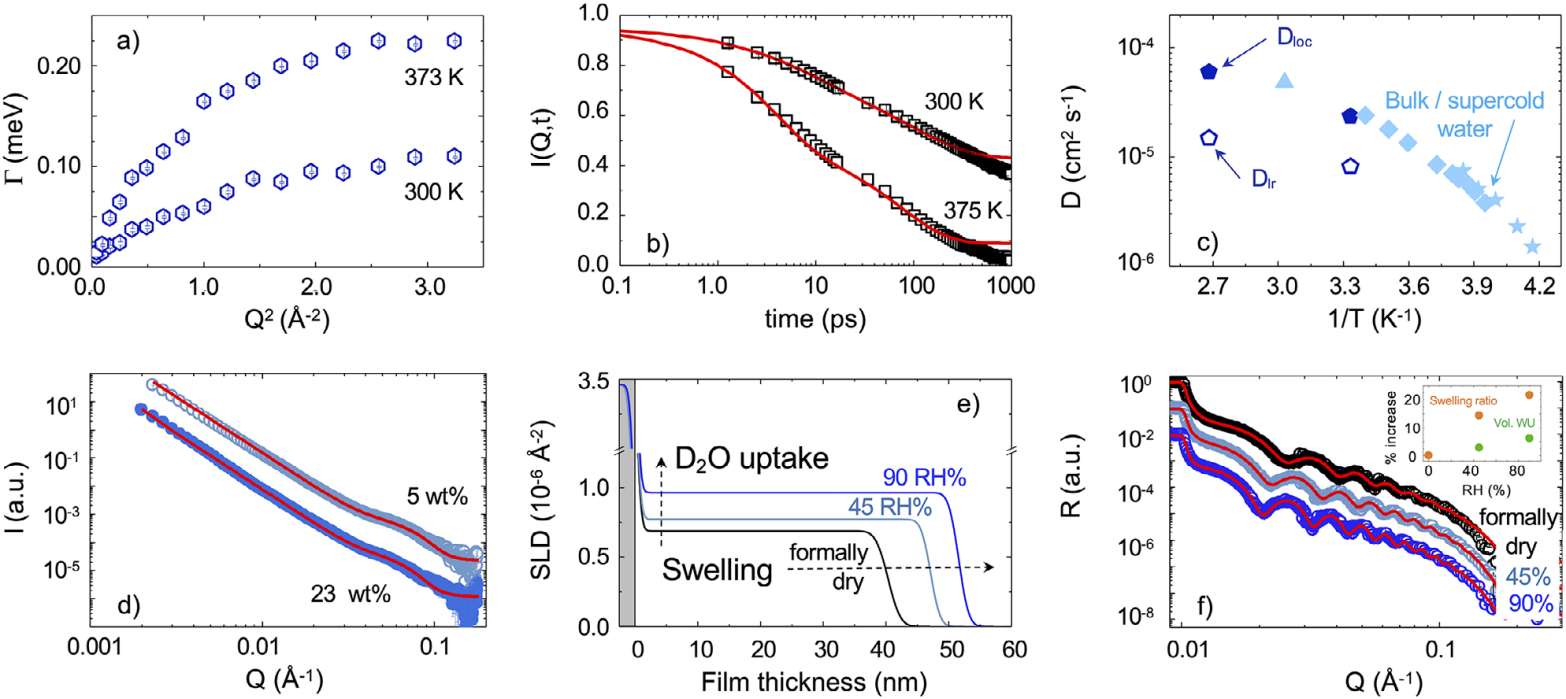
a) Linewidth HWHM (*Γ*) dependence on *Q* ^2^ measured for the 32 wt.% hydrated polymer (at 300 and 373 K). The error bars correspond to the fitting uncertainties of the linewidth. b) Scattering profile in the time domain for 32 wt.% hydrated polymer (at 300 and 373 K), and *Q*  =  1.4 Å^−1^. The global fit (red continuous curve) is overlaid across all datasets on the data points (black). c) Diffusion coefficients of the examined proton dynamic mechanisms (*D_loc_* = filled pentagon*, D_lr_* = open pentagon, light blue = literature of bulk water [49]) obtained from the QENS analysis. d) SAXS of polymer (blue circles) and model fit (red line) using Gaussian peak and Porod slope functions. e) Scattering length density (SLD) profiles for polymer thin film swelling vs relative humidity (RH). f) Reflectivity data with model fit (red line); extrapolated volumetric water uptake (WU) and swelling ratio of the polymer film from SLD profiles are presented in the inset.

To further examine the impact of hydration on membrane performance, the intermediate scattering function (*I(Q,t)*), covering a timescale of 1 ps to 1 ns, was fit to the Gaussian model (Figure 6a–c) to extract diffusion coefficients for two non‐exchangeable populations: localized (*D_loc_*) and long‐range (*D_lr_*). At 32 wt.% water uptake, hydrating water formed bulk‐like water channels with diffusion coefficients increasing from 2.35 to 5.97 × 10^−5^ cm^2^ s^−1^ and from 8.13 to 1.49 × 10^−6^ cm^2^ s^−1^ going from 300 to 373 K for *D_loc_* and *D_lr,_* respectively. Four slow protons associated with the methyl group in MsO^−^ and the protonated site on DAMAH^+^ were still observed.

Small‐angle X‐ray scattering (SAXS, Figure 6d) reveals a weak peak with ∼16 nm correlation length and large polydispersity, believed to be due to the microphase separation of large diffuse anions and cation/backbone rich phases. Peak polydispersity decreased as water uptake increased, signifying water‐induced microphase reorganization. Hydrated chain equilibration transforms the macromolecular architecture from confined ionic cages, where rapid local proton hops occur, into lower‐energy, uniform configurations where percolating hydrogen‐bond networks provide macroscopic conductivity.

Relative humidity‐dependent neutron reflectivity highlighted a significant non‐linearity between water uptake and water‐induced swelling (Figure 6e,f). Water forms low‐density solvation shells around the highly mobile, diffuse, and hydrophilic MsO^−^ anion, leading to matrix rearrangement with greater expansion than expected from bulk water. Solvation increases internal osmotic pressure and reduces ion‐ion pairing, removing physical cross‐linking density and expanding the polymer matrix. As water plasticizes the polymer, it also stabilizes the anion and reduces coordination with the amine, allowing chain rotations required for proton hopping geometries. Due to the hydrophilic nature of MsO^−^ and equivalence in pKa of MsOH and H_3_O^+^, the conductive pathway is formed homogeneously from water and anion, and under hydrated conditions the proton conduction is complemented by conventional Grotthuss and vehicular mechanisms.

## 3 Conclusion

In this work, we resolve the molecular origin of fast proton transport in [poly(DAMAH^+^ MsO^−^)], a promising class of protic poly(ionic liquid) membranes for high‐temperature electrochemical applications. Proton transport in poly(DAMAH^+^ MsO^−^) is strongly governed by both temperature (thermally activated dynamic transitions) and hydration (plasticization that lowers the effective dynamic constraints of the polymer matrix). By integrating multi‐resolution QENS with complementary PGF NMR, SAXS, and reflectometry, we reveal the complete dynamical landscape and directly observe the hierarchy of motions that enable long‐range proton conduction in the solid state. Fixed‐window activation energy analysis and temperature‐dependent QENS relaxation times revealed a polymer γ‐relaxation process at ∼290 K. This process was spatially resolved as the flipping of the DAMAH^+^ cationic unit rotating around the polymer axis, permitting hydrogen bond reorganization that facilitates a low‐barrier hopping pathway (*E_a_* ∼4.6 kJ mol^−1^) between MsO^−^ anions and ammonium sites. These findings are consistent with a polymer‐correlated Grotthuss‐type hopping mechanism, underlying the anhydrous macroscopic conductivity of these materials. Water uptake plasticizes the polymer, even at a low 5 wt.% water uptake, accelerating segmental relaxations and lowering the activation temperature for proton hopping. Higher hydration produces percolating hydrogen‐bond networks that supplement transport via conventional Grotthuss and vehicular mechanisms, while simultaneously solvating the diffuse and mobile anion. An additional transition in polymer dynamics is identified at ∼340 K, assigned as the glass transition, which induces a marked increase in long‐range polymer and proton transport, observed in NMR, and contributes to high conductivity at fuel cell operating temperatures. These insights resolve quantitative structure‐dynamics‐conductivity relationships and the long‐standing question of how solid polymers achieve liquid‐like proton mobility. This molecular‐level picture provides design principles for next‐generation solid proton conductors that combine high conductivity, mechanical robustness, and thermal resilience for intermediate‐ and high temperature‐PEMFCs and related electrochemical technologies.

## 4 Materials and Methods

### 4.1 Materials

Diallylmethylamine (DAMA, 98%) was bought from Acros Organics. Methanesulfonic acid (MSA, 99%) and thermal initiator 2,2′‐azobis(2‐methylpropionamide)dihydrochloride (AIBA) were purchased from Sigma–Aldrich. All reagents were employed with no further purification.

### 4.2 Preparation of [poly(DAMAH^+^ MsO^−^)]

Protic ionic liquid monomer, DAMAH^+^ MsO^−^, and its polymer were synthesized as previously reported [31]. The protic monomer was synthesized by an acid‐base reaction employing diallylmethylamine (DAMA) and methanesulfonic acid (MSA). The acid was added dropwise slowly to DAMA under agitation in a ratio 1:1 mol and left under agitation for 24 h to form the protic ionic liquid. One H^+^ is exchanged from the acid to the monomer DAMA, forming the protic ionic liquid DAMAH^+^ MsO^−^. Later, a free radical thermal polymerization was carried out: The monomer (30 wt.% in water) and the thermal initiator AIBA (3 wt.% in water) were first dissolved in the aqueous solution, which was subsequently deoxygenated for 30 min. Polymerization was then carried out at 65°C for 24 h under stirring.

After completion of the reaction, the resulting protic polymer was purified by dialysis to remove unreacted monomer. The purified poly(DAMAH^+^ MsO^−^) aqueous solution was dried under vacuum at 80°C for 48 h to obtain the final solid polymer. The chemical structure and successful polymerization of the orange dried polymer were confirmed by ^1^H NMR spectroscopy (Figure S6), confirming the classic cyclopolymerization mechanism of the diallylammonium monomer type, achieving the chemical structure of the protic polyIL observed in Figure 1a. Finally, a solvent‐casting process was carried out in order to obtain a protic polyIL membrane. An aqueous solution of poly(DAMAH^+^ MsO^−^) was cast into a silicon mold, and a two‐step drying process was carried out: a first step of 24 h at 80°C and a second step of 24 h at 80°C under vacuum to ensure total removal of humidity.

### 4.3 Thermal Gravimetric Analysis (TGA)

TGA measurements (Figure S7a,b) were carried out on a Waters TA Instruments Discovery TGA 5500 on ∼20 mg of sample under a nitrogen atmosphere (50 mL min^−1^) using a high‐temperature platinum pan, at a heating rate of 10°C min^−1^ from 30 to 600°C. The onset of degradation was obtained from the intersection of two tangents.

### 4.4 Water Uptake (WU) and Swelling Ratio (SR)

Prior to the measurements, samples were dried under vacuum at 60°C for 24 h to obtain their dry mass (W_d_). The dried samples were subsequently exposed to ambient conditions (Figure S7b). After set time intervals, the hydrated mass (W_w_) was recorded. WU (%) was calculated using: WU (%) = [(W_W_‐W_d_)/W_d_]×100%.

### 4.5 Differential Scanning Calorimetry (DSC)

DSC measurements were performed on a Waters TA Instruments Discovery DSC 2500 equipped with the liquid nitrogen pump accessory (LN2P). The samples were analysed under a helium atmosphere (flow rate 25 mL min^−1^) by cycling between ‐50 and 160°C. Around 10 mg of polymer was weighed out on a 6 d.p. balance (Sartorius Quintix 35–15) into a TA Tzero aluminium pan.

### 4.6 Electrochemical Impedance Spectroscopy (EIS)

EIS was employed to determine the ionic conductivity of dried pure poly(DAMAH^+^ MsO^−^) in an Autolab 302 N potentiostat/galvanostat at different temperatures (100–30°C) with an equilibration time of 20 min at each temperature before measurement to ensure equilibration. The sample was placed between two stainless steel electrodes (surface area of 0.5 cm^2^), and the thickness was kept constant using a 150 µm PTFE spacer. The measurements were obtained in the range between 300 KHz to 1 Hz, with a perturbation amplitude of 20 mV.

### 4.7 1H Nuclear Magnetic Resonance (NMR)

^1^H‐NMR spectra were recorded in a Bruker Avance DPX 300 at 300.16 MHz, using deuterated water (D_2_O) as solvent at room temperature for the analysis of the protic polyIL.

### 4.8 Pulsed Field Gradient (PFG) NMR

The ^1^H diffusion coefficients of dry and humid protic poly(DAMAH^+^ MsO^−^) were obtained using PFG‐NMR on a Bruker Avance III 300 MHz wide‐bore NMR spectrometer equipped with a 5 mm Diff 50 pulsed field gradient probe. The sample temperatures were calibrated with lead nitrate using the method described in previous studies [55].

The anhydrous samples were sealed into a 4 mm solid‐state NMR rotor inside the glovebox, which was then inserted into a standard 5 mm NMR glass tube for experiments. For hydrated samples, the 4 mm solid‐state NMR rotor was exposed outside the glovebox at a relative humidity of 75% for 12 h. A stimulated echo pulse sequence was used to measure both ^1^H diffusion coefficients. The typical gradient pulse duration was 10 ms, and the gradient strength was varied on a log scale, 32 steps between 1 and 2500 G cm^−1^. The diffusion time was 100 ms, the recycle delay was 2 s, and 16 scans were accumulated for ^1^H spectra. The diffusion coefficients were calculated from fitting of the NMR signal attenuation curve to the Stejskal‐Tanner equation and were used to determine the diffusion behavior of the protic cation backbone and labile proton species [56].

### 4.9 Quasi‐Elastic Neutron Scattering (QENS)

Polymer samples of varying water uptake (H_2_O and D_2_O) were achieved by weighing samples after subjecting them to the conditions below:

- dry: vacuum drying for 48 h at 60°C,
- 5 wt.%: ∼10 min exposure at 40% relative humidity,
- 23 wt.%: ∼2 h exposure at 40% relative humidity,
- 32 wt.%: ∼2 h exposure at 98% relative humidity

Hydrated samples were wrapped in Al foil and loaded into thin‐walled annular Al cans that were hermetically sealed with Indium wire. Quasi‐elastic neutron scattering was performed on the BASIS spectrometer at SNS (US) using an Energy resolution (E_res_) of 3.5 µeV and the IN5 spectrometer at Institut Laue Langevin (France) using an E_res_ of 80 µeV [57, 58]. Measurements were performed across the T range 2–373 K, and room temperature measurements were obtained before and after 373 K exposure to determine thermal stability.

Raw data were normalized to the neutron flux and detector efficiency fluctuations against a vanadium reference. The double differential cross section was converted into the corresponding dynamic structure factor *S(Q,ω)*. Data analysis was carried out directly on the *S(Q,ω)* spectra using Mantid [59], DAVE [60], or Origin 2019b. QENS data containing different contributions operating over different timescales were analyzed by fitting Lorentzian components, whose width and intensity inform on the nature of the dynamics. All plots representing the scattering profile analyses (*S(Q,ω) vs ΔE*) comprise the following components:

- Elastic contribution (delta function convoluted with *E_res_* function) obtained from the sample at ∼2 K.
- Polymer contribution (*Q*‐independent component).
- Ion‐hopping (*Q*‐independent component).
- Water diffusion (*Q*‐dependent component, where present).
- Flat background (accounting for mainly vibrational excitations that occur faster than the observational timescale).
- Global fit represented as red continuous curve.
- Experimental data points presented as black symbols.

### 4.10 Elastic Incoherent Structure Factor (EISF)

*Q*‐independence of the Lorentzian linewidth *Γ* of the dry polymer confirms the presence of only vibrational and rotational relaxation motions. The geometry of the motion, and thus the associated EISF profile, was constructed based on the chemical structure of the polymer repeat units, shown in Figure 4d. After fitting the total intensity with the above Lorentzian model, elastic *I_el_* (*Q*) and quasi‐elastic *I_qe_* (*Q*) contributions can be used to calculate the EISF by:

$$EISF\left(Q\right)=\frac{I_{el}\left(Q\right)}{I_{el}\left(Q\right)+I_{qe}\left(Q\right)}$$

The comparison of this experimental value with the theoretical EISF makes it possible to access the fraction of mobile protons, Φ*_mobile_*. Best agreement with experimental data was found for a model considering of three proton motions: i) jump rotation model between three equidistant sites on a circle with a radius, r, ($A_{0}=\frac{1}{3}\;\left[1+2j_{0}\left(Qr\sqrt{3}\right)\right]$), ii) jump among two equivalent sites ($A_{0}=\frac{1}{2};$ [1+j $j_{0}^{2}$ (Qd)]); where d is the jump distance and iii) out‐of‐plane partial rotation, or a ‘flip’, between two positions of distance d, and populations *p*_1_ and *p*_2,_ (*A*_0_ = [*p*_1_ *p*_1_ + *p*_2_ *p*_2_ + 2*p*_1_ *p*_2_ *j*_0_(*Qd*)] . *j*_0_ is the spherical Bessel function of the first order [47].

### 4.11 Elastic and Inelastic Fixed Window Scans (EFWS/IFWS)

Elastic experiments were performed to follow the evolution of both elastic and quasi‐elastic signal over a temperature scan while heating (1 K min^−1^) from 2 to 393 K. The proton mobility characteristics by measuring deviations from a reference position. Assuming harmonic and isotropic vibrational oscillations, the temperature dependence of the mean square displacement (*m.s.d., <u^2^>*) was obtained following the Debye‐Waller factor (DWF),

$$\frac{I_{inc_{elastic}}\left(Q,T\right)}{I_{inc_{elastic}}\left(Q,T_{min}\right)}=\exp\left(-\frac{1}{3}Q^{2}\left(\langle u^{2}\rangle -{\langle u^{2}\rangle }_{T_{min}}\right)\right)$$

Due to vibrational anharmonicity, the data deviate from the Debye‐Waller approximation as *T* is increased, especially at higher *Q*. The onset of localized hopping or polymer dynamics also begins to contribute to *<u^2^>* at higher temperature, thus *d<u^2^>/dT* is obtained <100 K.

IFWSs were acquired by integrating an arbitrarily chosen energy range (having an integration width equivalent to *E*_res_ – 3.5 µeV for BASIS). Estimation of the activation energy, *E_a_*, associated with the dynamic process was obtained from the temperature variation by following equations,

$$I_{\omega_{off}}^{IFWS}\left(T\right)\propto \frac{B}{\pi }\left(1-A_{0}\left(Q\right)\right)\frac{\tau \left(T\right)}{1+\omega_{off}^{2}\tau {\left(T\right)}^{2}}$$

$$\tau \;\left(T\right)=\tau_{0}\;exp\left[-\frac{E_{a}}{KT}\right]$$

$$T_{max}=\frac{E_{a}/KT}{\ln\left(\frac{1}{\omega_{off}\tau_{0}}\right)}$$

where *A_0_* is the EISF, *B* is a constant which considers the resolution convolution factor, *τ* is the relaxation time where a departure from quasi‐elastic intensity is observed (ps), and K is the Boltzmann constant [37].

### 4.12 Small and Wide Angle X‐Ray Scattering (SAXS/WAXS)

Membranes were exposed to ambient humidity until reaching specified water uptake and analyzed using SAXS and WAXS on the I22 SAXS Pilatus detector at Diamond Light Source, UK. Scattering profiles were acquired at 12.5 keV (0.689 Å) with a sample‐to‐detector distance of 2.87 m; this configuration allowed for investigating a *Q*‐range from 0.0025 to 5 Å^−1^. Data were processed on DAWN software [61] and fit to a model summing a Gaussian peak and Porod slope using SASview software [62].

### 4.13 Neutron Reflectivity (NR)

The aqueous polymer dispersion (1 wt.%) was spin‐coated onto atomically smooth, planar crystalline silicon blocks at 1800 rpm for 2 min and completely dried in a vacuum oven at 60°C for 24 h before measurements. NR was performed at the Institute Laue Langevin (France) using the D17 reflectometer [63, 64]. Polymer thin films (40–55 nm) were loaded in a sealed oven with humidity control provided by exposure to combined dry and hydrated (D_2_O) N_2_ flows to achieve the desired temperature and humidity. Data reduction and analysis were performed using Mantid [59] and Refnx [65]. NR profiles at the air/solid (Silicon) interface were modelled using a single uniform polymer layer and a native SiO_2_ layer to generate the SLD profile.

## Author Contributions

**Antonela Gallastegui**: writing – review and editing, data curation, validation, formal analysis. **Yuliana Pairetti**: data curation, visualization. **Aurelie Gueguen**: conceptualization, methodology, validation, supervision, writing – review and editing, data curation. **Maria Forsyth**: methodology, formal analysis, supervision, writing – review and editing, data curation. **Jacques Ollivier**: methodology, supervision, data curation, writing – review and editing. **Andrew Seel**: methodology, validation, writing – review and editing. **Zixuan Yu**: data curation, writing – review and editing, visualization. **David Mecerreyes**: conceptualization, methodology, validation, formal analysis, supervision, writing – review and editing. **Fabrizia Foglia**: conceptualization, investigation, funding acquisition, writing – original draft, writing – review and editing, methodology, data curation, supervision, resources, validation, visualization, formal analysis. **Victoria Garcia Sakai**: methodology, validation, writing – review and editing. **Bob C. Schroeder**: investigation, validation, formal analysis, writing – review and editing. **Keenan Smith**: validation, writing – review and editing, data curation, formal analysis.

## Conflicts of Interest

The authors declare no conflicts of interest.

## Supporting information




**Supporting File**: advs77430‐sup‐0001‐SuppMat1.pdf.

## Data Availability

The data that support the findings of this study are available from the corresponding author upon reasonable request. The original neutron data are available at https://doi.org/10.5291/ILL‐DATA.9‐11‐2016; and https://doi.org/10.5291/ILL‐DATA.9‐11‐2087.
