# Supplementary material for: Correlated Polymer‐Proton Hopping Enables Water‐Independent Fast Proton Transport in Poly(Ionic Liquid) Membranes
Source: Adv Sci (Weinh). 2026 Aug 29:e77430. Online ahead of print. doi: 10.1002/advs.77430 (PMC13525415; doi:10.1002/advs.77430)
Supplement: Supplementary file 1 [file ADVS-9999-e77430-s001.pdf]

Supplementary Material

Correlated polymer-proton hopping enables water-independent fast proton transport in poly(ionic liquid) membranes

*Keenan Smith†, Antonela Gallastegui†‡, Zixuan Yu, Yuliana Pairetti, Andrew Seel, Jacques Ollivier, Victoria Garcia Sakai, Bob C. Schroeder, Maria Forsyth, Aurelie Gueguen, David Mecerreyes, and Fabrizia Foglia**

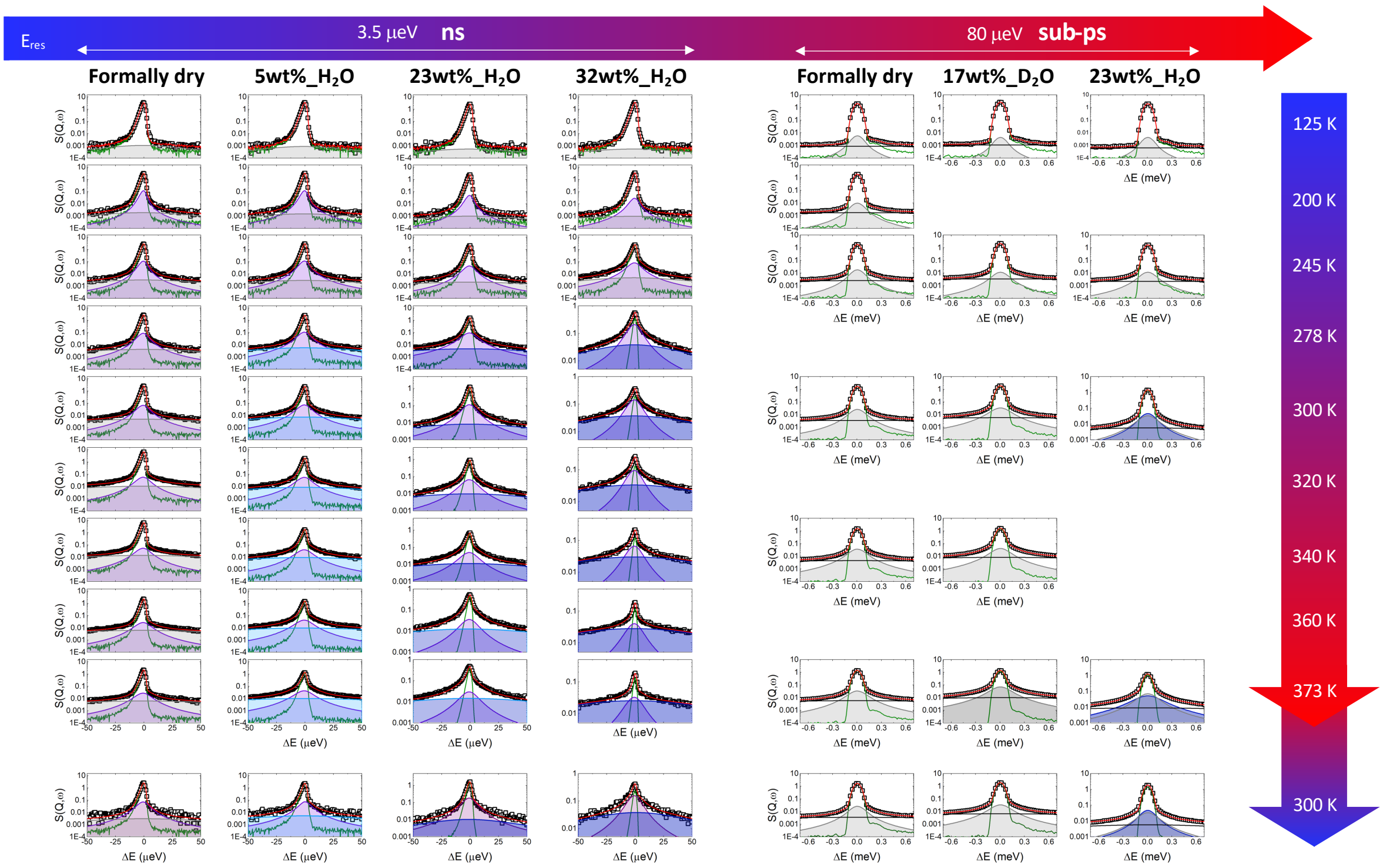

Figure S1. *Q*-summed QENS experimental data and fitting profiles for [poly(DAMAH$^+$ MsO$^-$)] at range of water uptake (wt%) in H_2O and D_2O at corresponding T at the two energy resolutions studied: E_{res} = 3.5 μeV (BASIS, left) and E_{res} = 80 μeV (IN5, right).

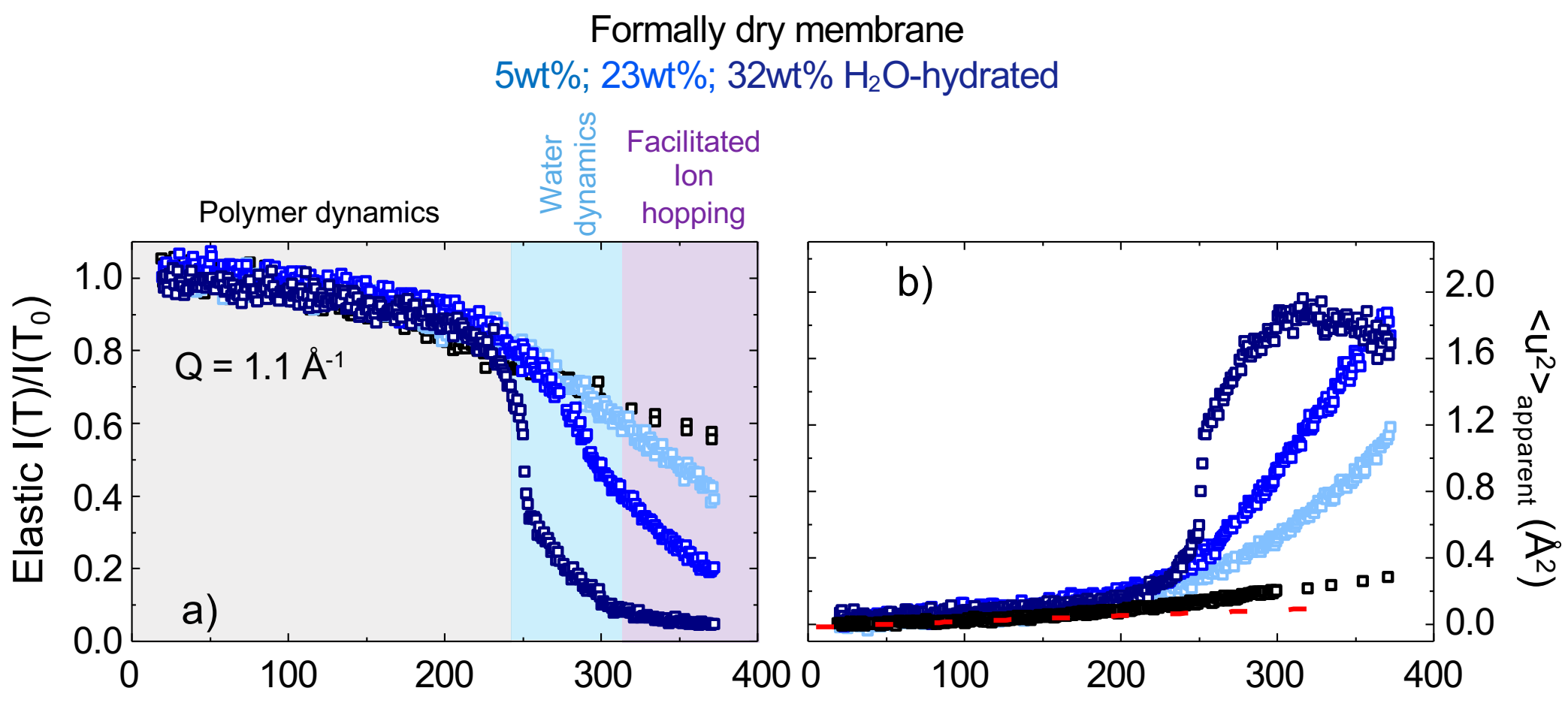

Figure S2. a) Elastic Fixed Window Scan (EFWS) and b) apparent mean square displacement (m.s.d., $<u^2>_{apparent}$) acquired on BASIS spectrometer for different hydration levels by integrating scattering profiles at 0 μeV ΔE [31].

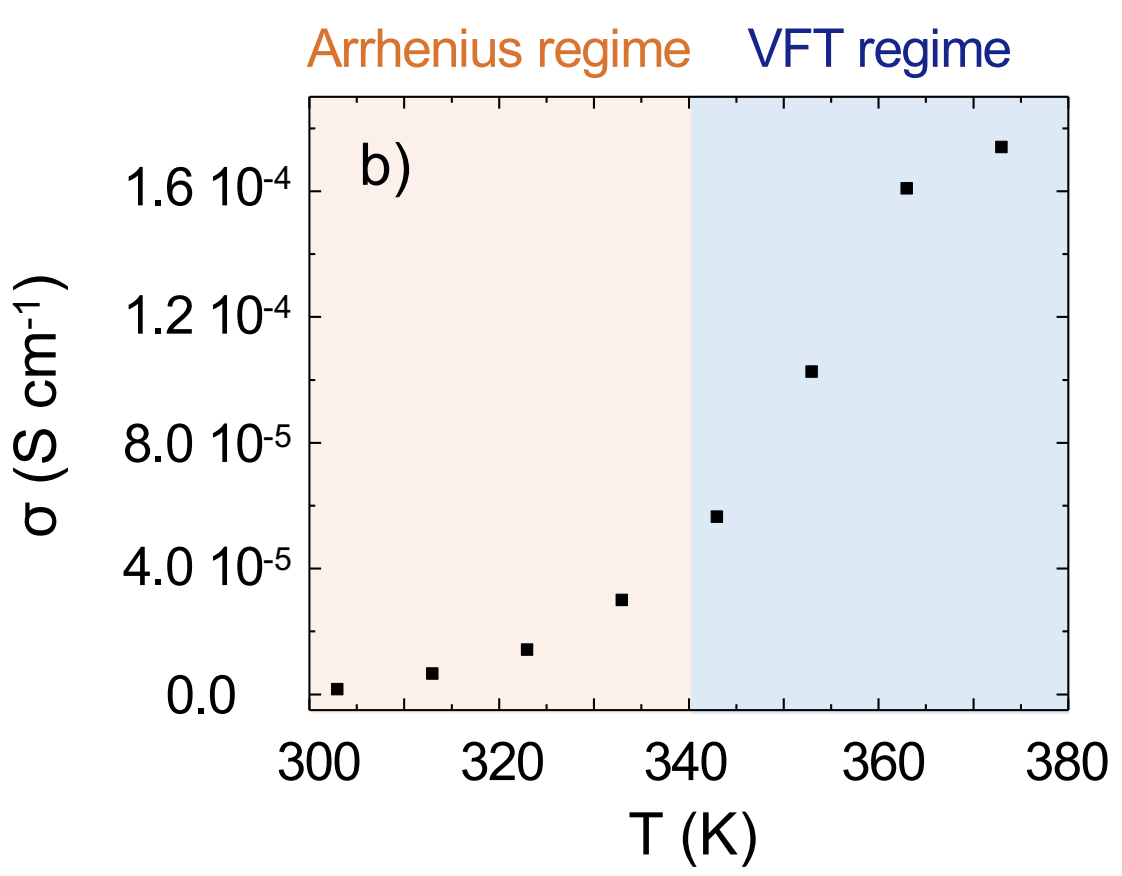

Figure S3. Ionic conductivity of poly(DAMAH$^+$ MsO$^-$) in the formally dry state as function of the temperature (30 - 100 ºC), determined by by electrochemical impedance spectroscopy (EIS).

Supplementary table 1. Activation energy calculated from ionic conductivity measurements using both Arrhenius and Vogel-Fulcher-Tammann (VFT) model.

**Temperature (K)**	**Equation**	**A**	**B (K)**	**T_0 (K)**	**R^2**	**Activation energy (kJ mol^{-1})**	**Formula**
**303 – 373**	$\sigma = A\exp(-E_a/RT)$	1.86×10^5				63.1	Slope $(-E_a/R) = -7584$
**303 – 333**	$\sigma = A\exp(-E_a/RT)$	1.77×10^8	0.981		0.981	81.1	Slope $(-E_a/R) = -9757$
**343 – 373**	$\sigma = A\exp(-B/(T-T_0))$	4.01×10^{-4}	40.0		0.988	115.2 (341)<br>94.8 (343)<br>45.1 (353)<br>27.0 (363)<br>18.3 (373)	$E_a(T) = (R \times B \times T^2)/(T-T_0)^2 - B$

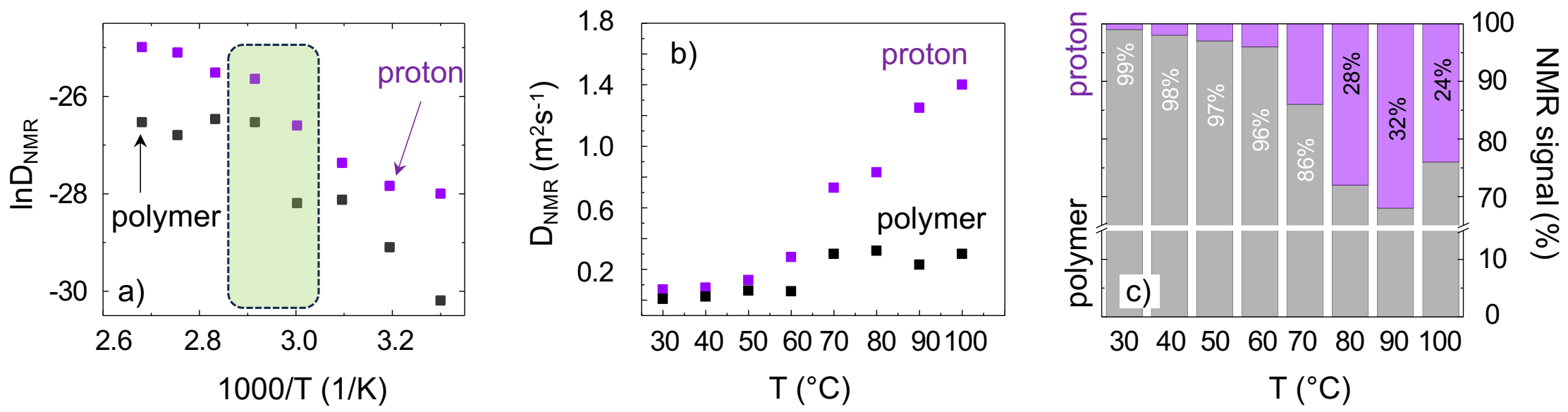

Figure S4. a) Diffusion coefficients of labile proton and polymer in poly(DAMAH$^+$ MsO$^-$) at 5wt% hydration obtained by ^{1}H PFG NMR from 30 to 100 ºC plotted a) logarithmically with green highlighting transition region and b) linearly. c) proportion of NMR signal for labile proton and polymer from 30 to 100 ºC.

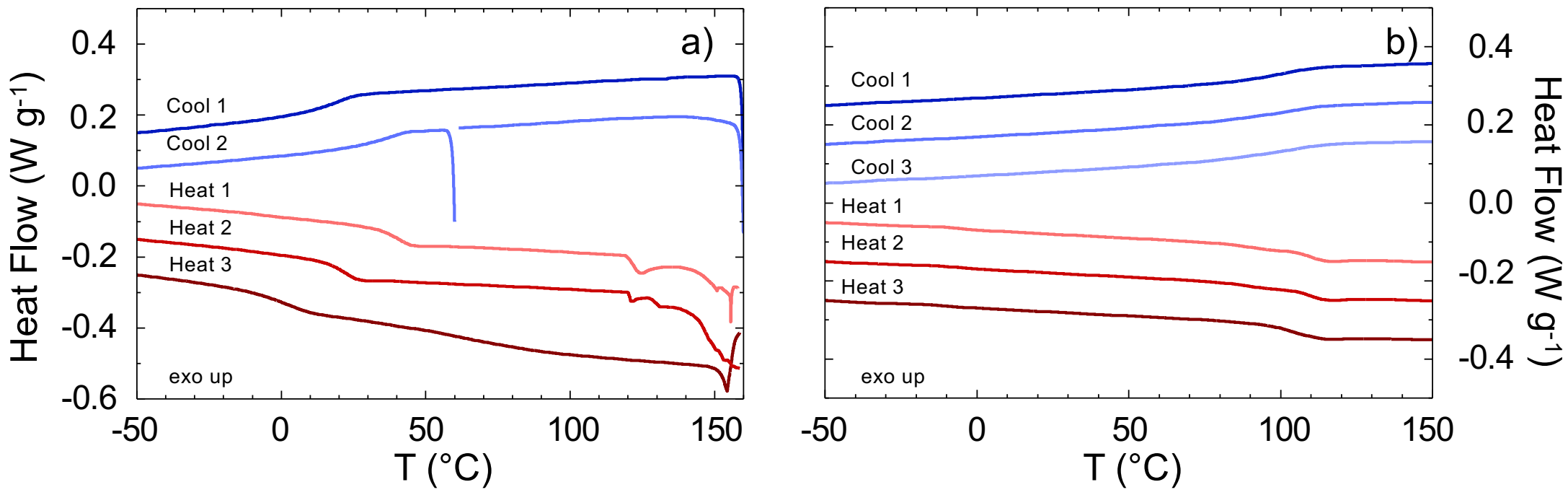

Figure S5. DSC thermograms of poly(DAMAH$^+$ MsO$^-$) recorded at 10 °C min^{-1}. **a)** Hydrated sample across three heating (Heat 1-3) and two cooling cycles (Cool 1-2), revealing a progressive upshift in T_g from ~0 to ~40 °C as water is driven off. **b)** Fully dehydrated sample (achieved by annealing at 200 °C for 300 min in the DSC cell) across three cooling and heating cycles (Cool/Heat 1-3).

Initially the DSC was performed on the 10% hydrated polymer sample to evaluate the effect of hydration on the material properties, particularly the DSC. During the first heating cycle of the hydrated sample the T_g was observed at 0 °C (**Figure S5a**). This depressed T_g is the result of the interplay of solvated water molecules with the polymer chain as commonly observed for hydrogels [66]. Upon further heating water evaporation was observed at around 155 °C. During the second heating cycle the *Tg* shifted to 21 °C as the polymer was now partially dehydrated. The appearance of additional sharp endothermic peaks from 110 °C onwards, provides further evidence for additional water evaporation and rapid evaporation of water vapour trapped inside the polymer sample itself or the DSC pan. To remove additional water from the polymer, the sample was cooled to 60 °C, assumed sufficiently high to be above the actual polymer *Tg*. The sample was annealed at this temperature for 480 minutes, before cooling down and subjecting the polymer to a third heating cycle. Once again the T_g shifted (40 °C), yet upon further heating additional water evaporated from the sample (endothermic transitions above 110 °C). Consequently, the sample was heated to 200 °C for 300 minutes, to ensure complete dehydration of the polymer sample. The samples were cooled to -50 °C and heated again to 160 °C, with no endothermic peaks observable above 100 °C, confirming complete dehydration of the poly(DAMAH$^+$ MsO$^-$). In the dehydrated polymer the T_g was observed at 107 °C, repeatedly over three cooling and heating cycles (**Figure S5b**), confirming that the hydration level of poly(DAMAH$^+$ MsO$^-$) has a profound impact on its thermomechanical properties.

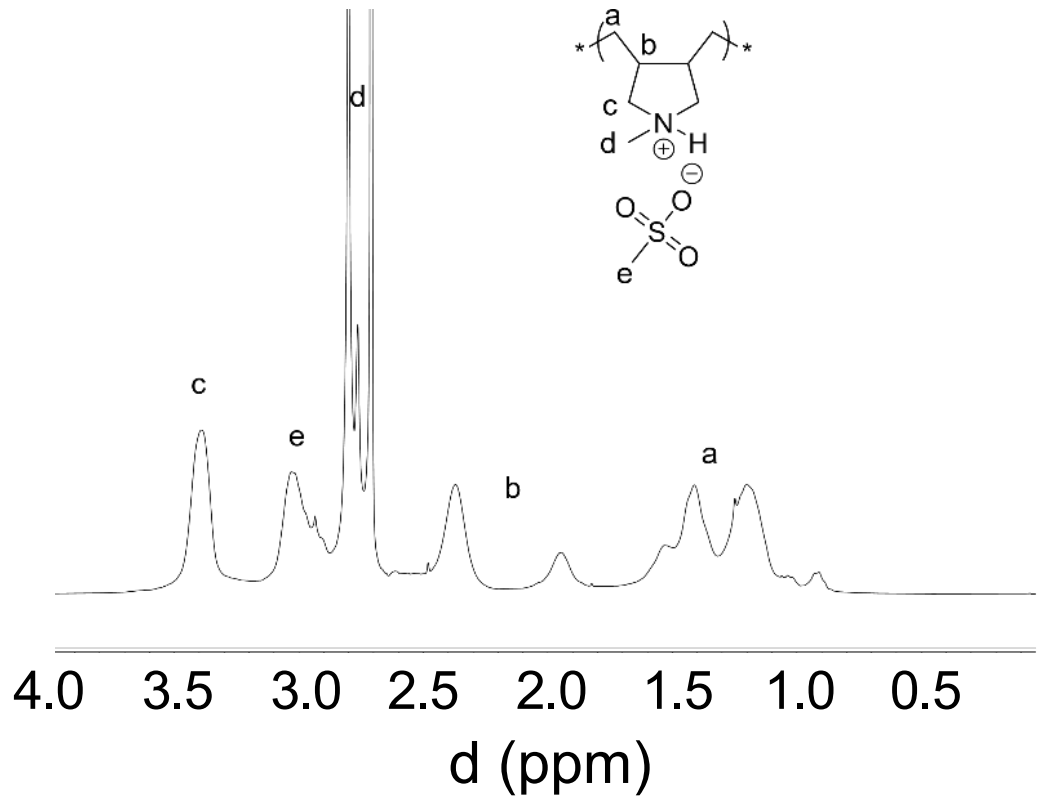

Figure S6. ^{1}H NMR spectrum of poly(DAMAH$^+$MsO$^-$) in D_2O.

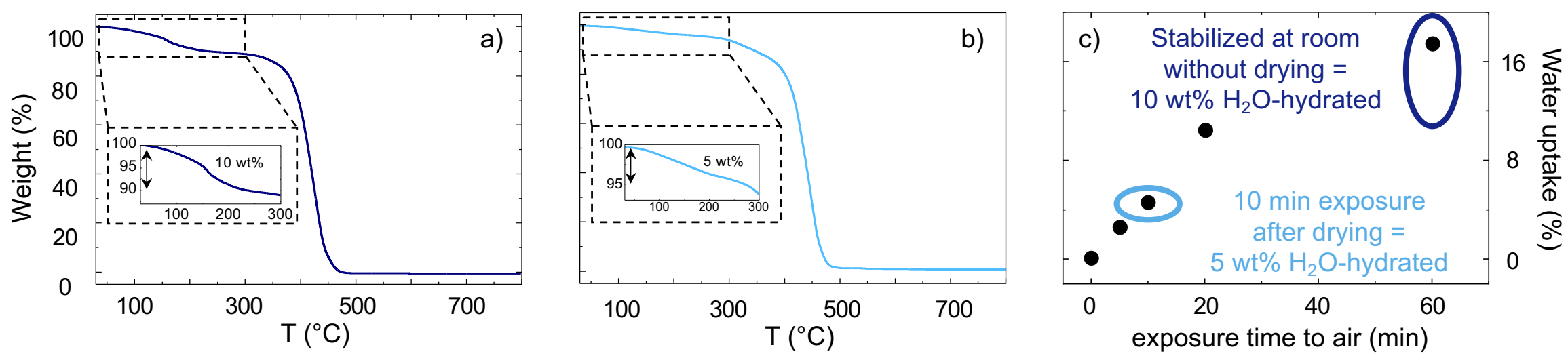

Figure S7. **a-b)** TGA of poly(DAMAH$^+$ MsO$^-$). The sample in panel a was loaded directly without prior drying, whereas the sample in panel b was dried overnight before measurement, illustrating the influence of residual water content on thermal stability. **c)** Time-resolved water uptake upon exposure to ambient humidity.

References

66. J.A. Bouwstra, M.A. Salomons-de Vries, and J.C. van Miltenburg, "The thermal behaviour of water in hydrogels," *Thermochimica Acta* 248 (1995): 319-327.
